# Treatment of brainstem and fourth ventricle lesions by the full neuroendoscopic telovelar approach

**DOI:** 10.1186/s40001-023-01460-5

**Published:** 2023-12-05

**Authors:** Long Zhou, Hangyu Wei, Zhiyang Li, Huikai Zhang, Ping Song, Li Cheng, Wenju Wang, Pan Lei, Qianxue Chen, Zaiming Liu, Hui Ye, Daofa Sun, Qiang Cai

**Affiliations:** 1 Department of Neurosurgery, Renmin Hospital of Wuhan University, No. 238, Jiefang Road, Wuchang District, Wuhan City, 430060 Hubei Province China; 2 Department of Critical Care Medicine, Eastern Campus, Renmin Hospital of Wuhan University, Wuhan, China; 3 Department of Neurosurgery, Xiantao First People’s Hospital of Yangtze University, No. 29, Middle Part of Mianzhou Avenue, Xiantao City, 433000 Hubei Province China

**Keywords:** Transcranial neuroendoscope, Telovelar approach, Brainstem lesion, Fourth ventricle, Cavernous hemangioma

## Abstract

**Objective:**

To explore the surgical techniques, advantages, and disadvantages of neuroendoscopic telovelar approach in the treatment of brainstem and fourth ventricle lesions.

**Methods:**

The clinical data of 5 patients treated by neuroendoscopic telovelar approach from March 2020 to March 2022 were analyzed retrospectively.

**Results:**

Among the 5 patients, there were 3 cavernous hemangiomas in pontine arm and 2 tumors in brainstem and fourth ventricle. All patients could successfully complete the operation, and 4 patients recovered well, other 1 patient discharged automatically for serious complications of other systems after the operation.

**Conclusion:**

The telovelar approach has gained popularity as a safe and effective strategy for lesions in fourth ventricular and brainstem. However, without removing the posterior arch of the atlas, it is difficult to enter the upper part of the fourth ventricle under a microscope. Transcranial neuroendoscopy can effectively compensate for the shortcomings of microscopy, whether used as an auxiliary measure for microsurgery or alone with proficient endoscopic techniques, it will provide greater application in minimally invasive surgery for fourth ventricle and brainstem lesions. By utilizing the excellent degree of freedom of transcranial neuroendoscopy, there is no need to open the posterior arch of the atlas, making the surgery more minimally invasive. However, the sample size of this study is small, and it was completed under the very mature neuroendoscopic technology of our team. Its general safety and practicality still require extensive clinical research validation.

## Introduction

Lesions located deep in the fourth ventricle and/or pontine tegmentum are challenges for neurosurgeons due to the limited working space and the complexity of the surrounding structures which include the medulla, lower cranial nerve nuclei, cerebellar peduncles, and posterior inferior cerebellar artery (PICA). Traditional surgery approach for this area involves splitting the inferior vermis to gain better direct access, which is also known as the transvermian approach. However, this approach can lead to postoperative disturbances of equilibrium and cerebellar mutism [1]. Operating through cerebellomedullary fissure by telovelar approach has also been used to reach this area without damaging inferior vermis which has been confirmed to be a reliable approach [2–4]. During the last 3 decades, all telovelar approaches have been performed under the microscope, and gradually found that it was difficult to access the upper fourth ventricle and pontine tegmentum [1, 5]. To date, no case of upper fourth ventricle and pontine tegmentum lesions resected by full neuroendoscopic telovelar approach was reported. Our early clinical research shows that transcranial neuroendoscopic has obvious advantages in retrosigmoid approach, which can make up for the deficiency of microscopic surgery [6]. In this clinical study, we tentatively used the full transcranial neuroendoscopic telovelar approach to remove the lesions of brainstem and fourth ventricle, and most patients recovered excellent.

## Data and method

### General data

In this study, the data of neurosurgery patients in our hospital from March 2020 to March 2022 were collected. The inclusion criteria were patients with treatment of brainstem and fourth ventricle lesions by the full neuroendoscopic in telovelar approach, including brainstem cavernous hemangioma, brainstem tumor, etc. The preoperative and postoperative imaging data, intraoperative neuroendoscopic video images and clinical manifestations were collected. A total of 5 patients with complete data were collected, including 1 male and 4 females, aged 3–67 years. The detailed clinical information is shown in Table 1.

**Table 1:** Clinical, surgical, and outcome data

Number	Gender	Age	Diagnosis	Location	Lesion volume (cm^3^)	The operation position	Neurological symptoms	
							Preoperative	Postoperative
1	F	62	Right pontine cavernous hemangioma hemorrhage	Pontine arm	1*1*1	Lateral and prone position	Dizziness with vomiting	Normal
2	F	54	Left pontine cavernous hemangioma hemorrhage	Pontine arm	2*1.4*1.2	Lateral and prone position	Intermittent headache and dizziness with numbness of the right limb	Right limb muscle strength grade 4
3	F	67	Right pontine cavernous hemangioma hemorrhage	Pontine arm	1.5*1.2*1.5	Prone position	Dizzy	Right mydriasis
4	M	3	Brainstem medulloblastoma	Brain stem and fourth ventricle	3.2*2.6*3.3	Lateral and prone position	Unstable walking, right muscle strength level 4	Coma, respiratory failure
5	F	32	Pilocytic astrocytoma	Pontine and fourth ventricle	0.8*0.8*0.8	Lateral and prone position	Dizziness with vomiting	Normal

### Surgical technique

**Fig. 1:**
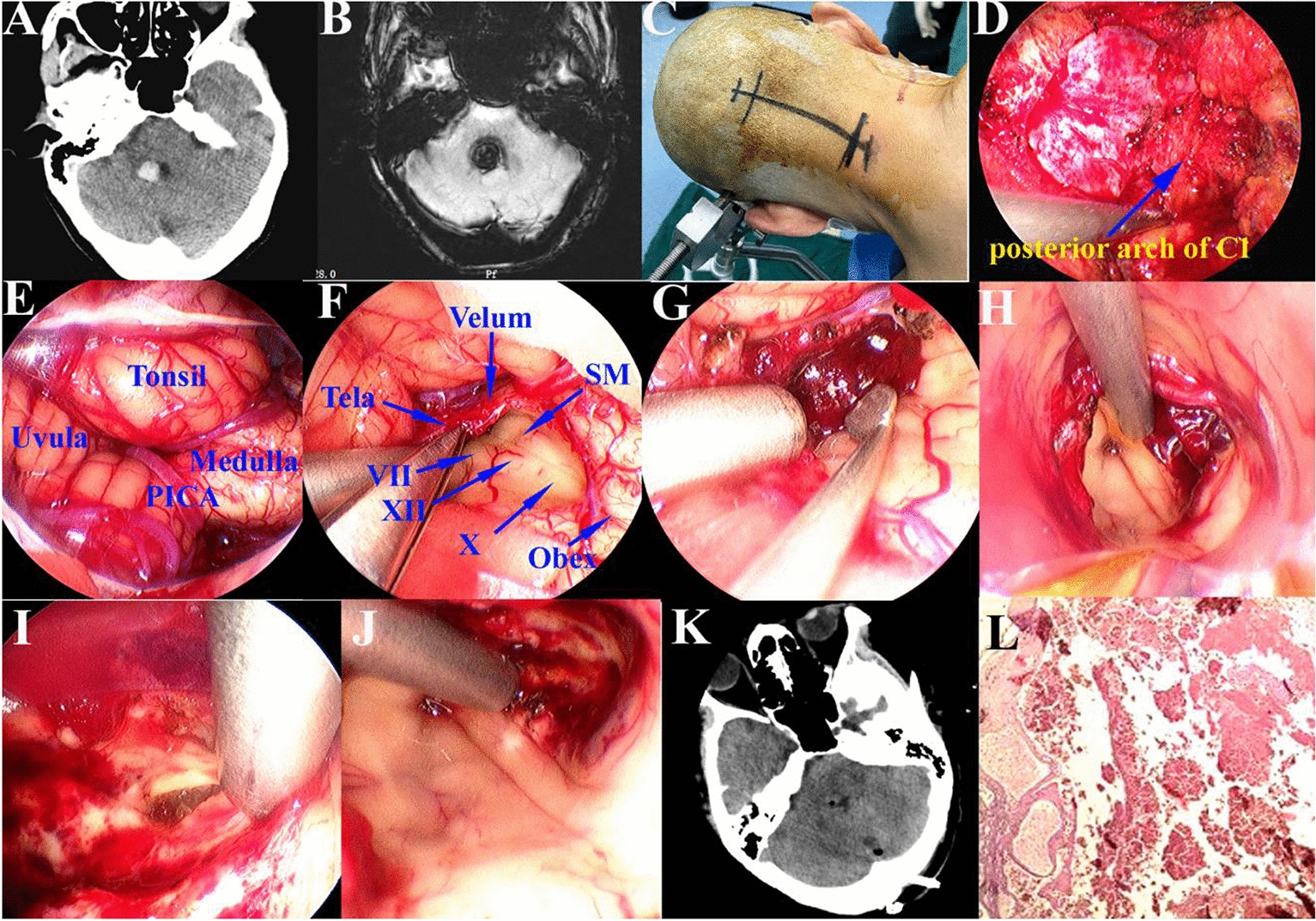
Resection a middle cerebellar peduncle and pontine tegmentum cavernous malformation by full neuroendoscopic telovelar approach. **A** CT imaging shown a small hematoma in the right fourth ventricle, middle cerebellar peduncle and pontine tegmentum. **B** MRI SWAN sequence suggested a cavernous malformation; **C** patient in the lateral prone position with the head fixed in a head-holder and slightly flexed; **D** a midline suboccipital craniotomy was performed, and the posterior arch of C1 was preserved; **E** under neuroendoscopic view, the inferior edge of the tonsils, uvula, PICA and obex was exposed. **F** The tela choroidea, inferior medullary velum and floor of the fourth ventricle were visualized and protected. **G** The hematoma in the fourth ventricle was exposed and removed. **H** The hematoma in the middle cerebellar peduncle was exposed and removed. **I** A small, cavernous malformation in the pontine tegmentum was identified and removed. **J** The cavity was checked after the operation. **K** Postoperative CT scan shown the hematoma was removed completely. **L** Histopathological examination revealed a cavernous malformation

**Fig. 2:**
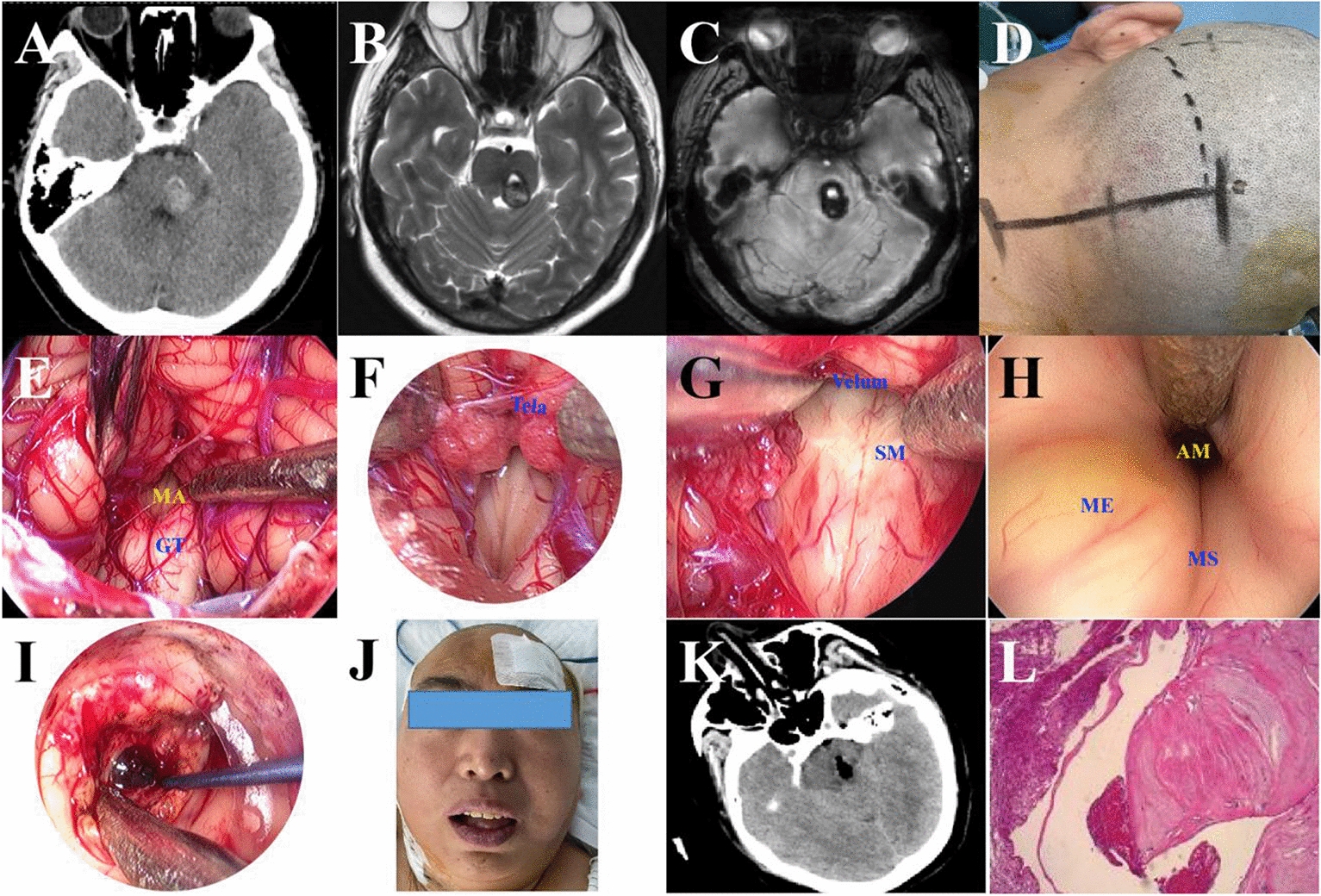
Resection a pontine tegmentum cavernous malformation by full neuroendoscopic telovelar approach. **A** CT imaging shown a small hematoma in the left pontine tegmentum. **B** T2-MRI shown irregular and heterogeneous signals in pontine tegmentum; **C** MRI SWAN sequence suggested a cavernous malformation; **D** head fixed in a head-holder and slightly flexed; **E** the median aperture of the fourth ventricle was exposed. **F** The tela choroidea, inferior medullary velum was visualized and protected. **G** The floor of the fourth ventricle was exposed. **H** The upper fourth ventricle floor was exposed, and the median eminence yellowing. **I** A small, cavernous malformation in the pontine tegmentum was identified and removed. **J** The patient recovered well after the operation. **K** Postoperative CT scan shown the hematoma was removed completely. **L** Histopathological examination revealed a cavernous malformation

**Fig. 3:**
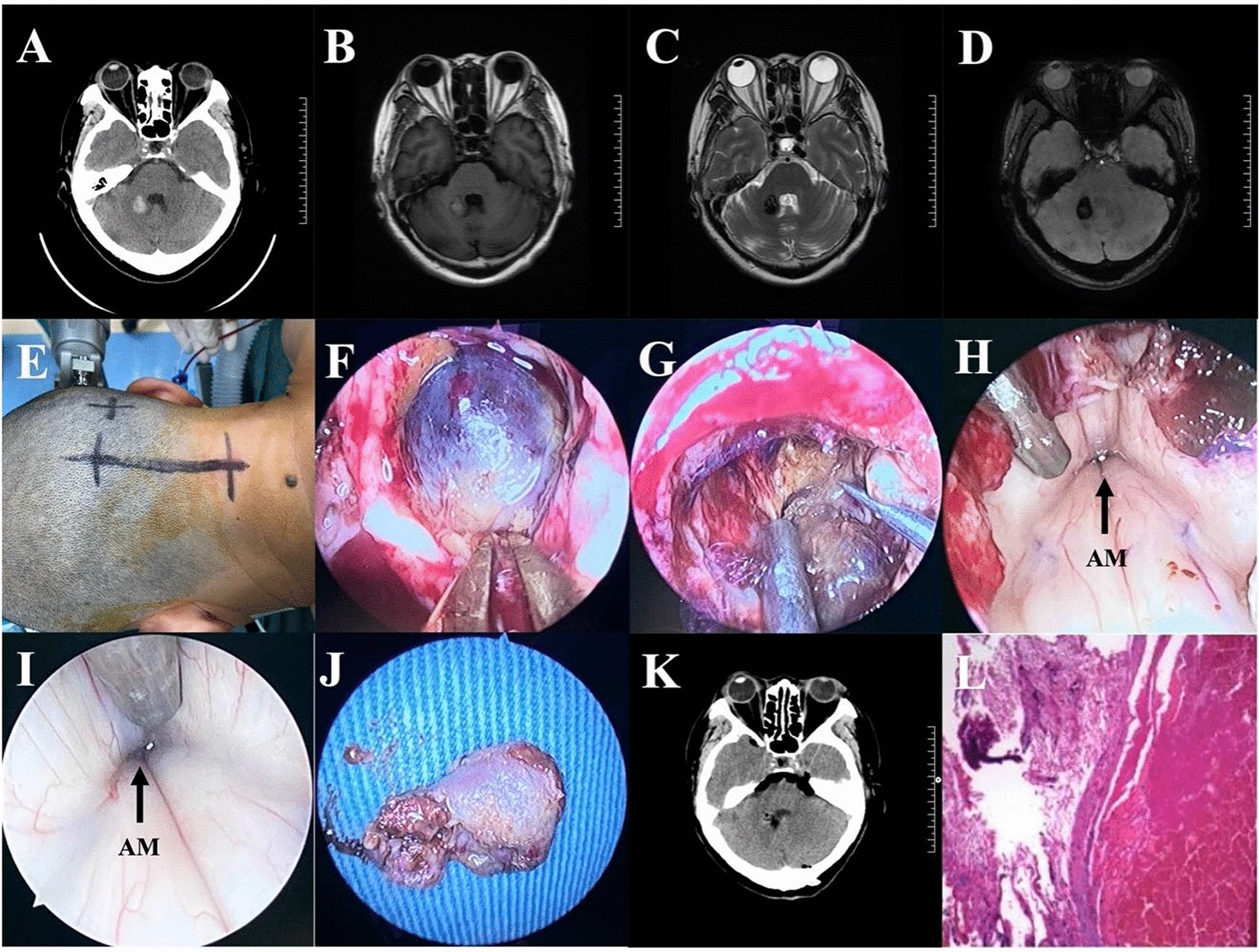
Resection a right pontine arm cavernous malformation by full neuroendoscopic telovelar approach. **A** CT imaging shown a small hematoma in the right pontine arm. **B**–**D** MRI showed a cavernous hemangioma with hemorrhage in the right pontine arm. **E** Head fixed in a head-holder and slightly flexed. **F** The lesion was seen under neuroendoscopy. **G** Resection of lesion under neuroendoscopy. **H**, **I** After lesion resection, neuroendoscopic exploration of the fourth ventricle showed the outlet of aqueduct of midbrain. **J** The complete lesion. **K** Postoperative CT scan shown the lesion was removed completely. **L** Histopathological examination revealed a cavernous malformation

**Fig. 4:**
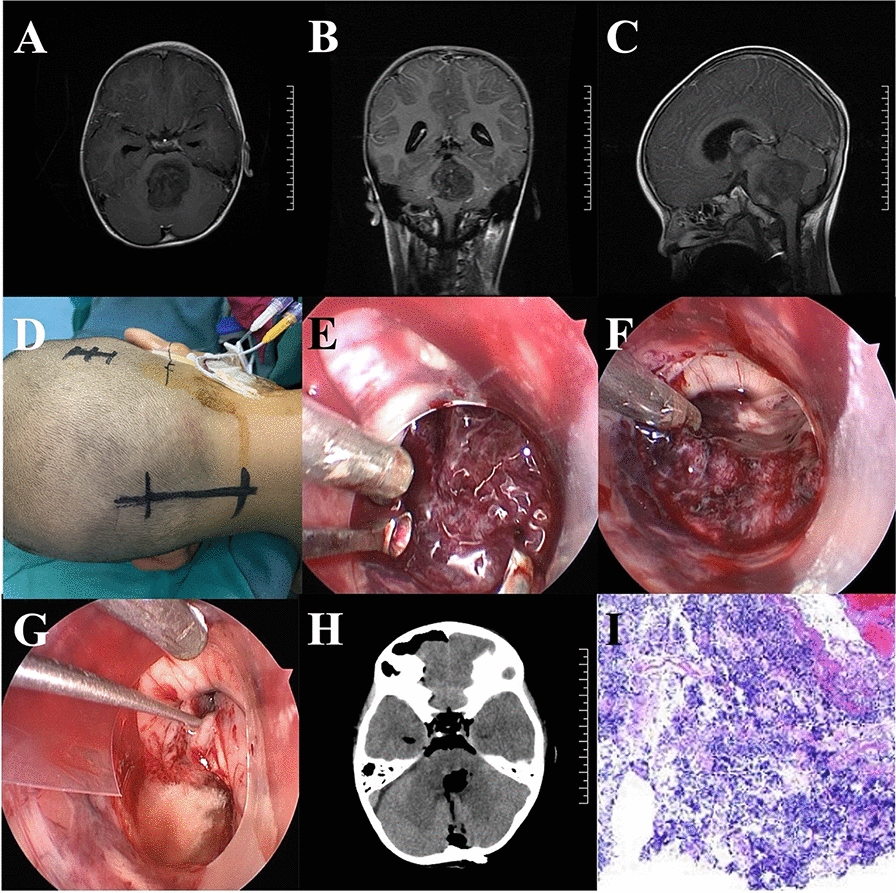
Total neuroendoscopic resection of brainstem medulloblastoma. **A**–**C** MRI showed space occupying lesions of brainstem. **D** Head fixed in a head-holder and slightly flexed. **E** The lesion was seen under neuroendoscopy. **F** Resection of lesion under neuroendoscopy. **G** The lesion was completely resected under neuroendoscopy. **H** Postoperative CT scan shown the lesion was removed completely. **I** Histopathological examination revealed a medulloblastoma

**Fig. 5:**
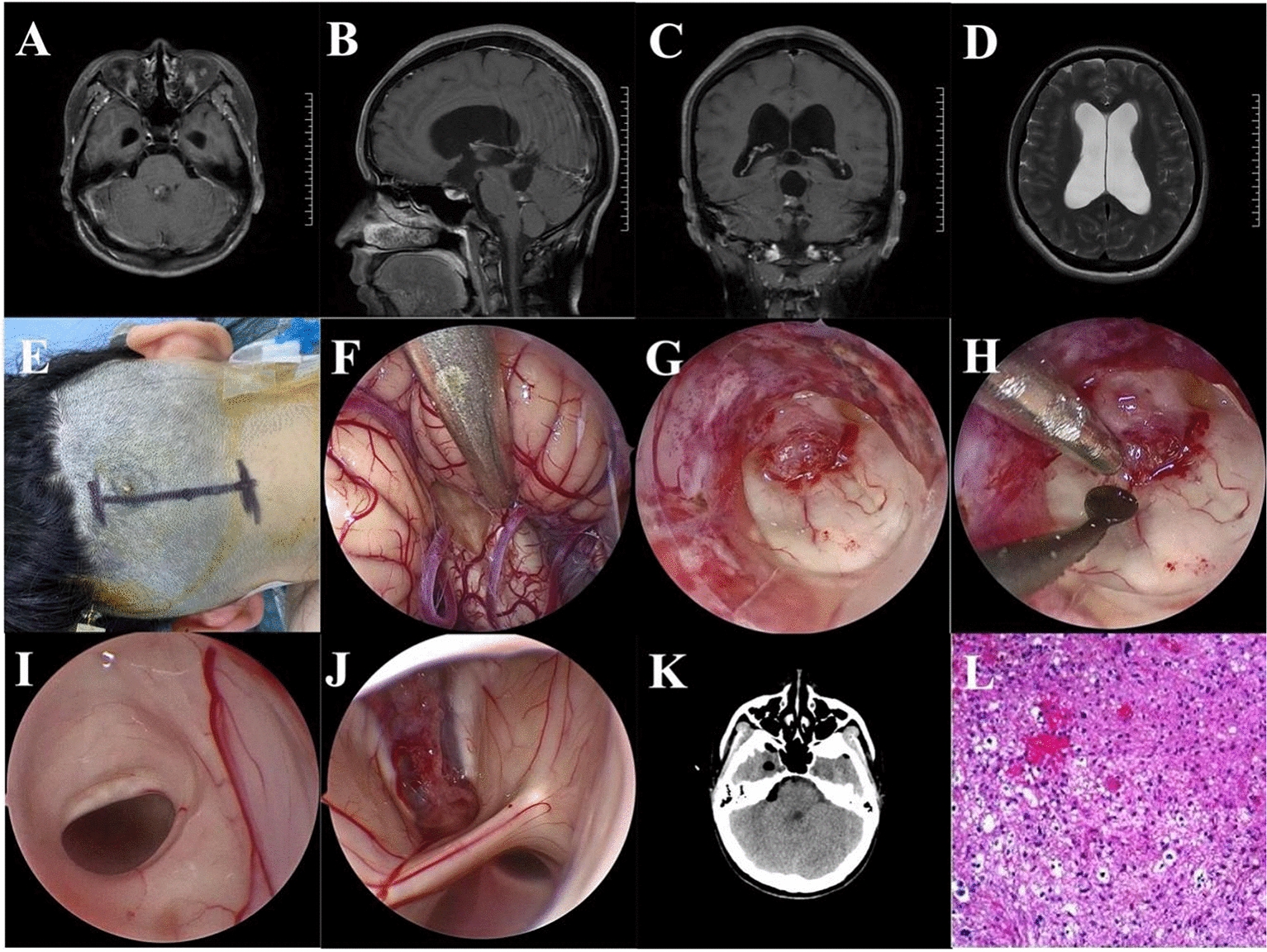
Total neuroendoscopic resection of astrocytoma of brainstem and fourth ventricle. **A**–**C** MRI showed space occupying lesions in the brain stem and the top of the fourth ventricle. **D** MRI showed supratentorial hydrocephalus. **E** Head fixed in a head-holder and slightly flexed. **F** The median aperture of the fourth ventricle was exposed. **G** The lesion was seen under neuroendoscopy. **H** Resection of lesion under neuroendoscopy. **I** Enlarged midbrain aqueduct outlet after hydrocephalus. **J** Exploration of the third ventricle via midbrain aqueduct using neuroendoscopy. K: Postoperative CT scan shown the lesion was removed completely. **L** Histopathological examination revealed a pilocytic astrocytoma. SM: stria medullaris; VII: facial colliculus; XII: hypoglossal triangle; X: vagal triangle; GT: gracile tubercle; MA: median aperture; MS: median sulcus; ME: median eminence; AM: aqueduct of midbrain; PICA: posterior inferior cerebellar artery

Surgical procedures were performed with the patient in the lateral prone position, the head fixed in a head-holder with slight flexion. A midline suboccipital craniotomy was performed to expose the craniovertebral junction, and the posterior arch of the atlas was preserved. Under neuroendoscopic view, a Y-shaped dural opening was made, and the inferior edge of the tonsils, uvula, PICA and obex were exposed (Figs. 1–5). The tonsil and uvula were elevated and retracted by a thin transparent endoport, and then the tela choroidea, inferior medullary velum and floor of the fourth ventricle were visualized and protected. Looking forward to the upper ventricle, the hematoma was identified and removed under the neuroendoscopy. With further access to the cerebellar peduncle and pontine tegmentum area, the residual hematoma in the cerebellar peduncle was cleared away, and a small, cavernous malformation in the pontine tegmentum was identified and removed (Figs. 1, 2 and 3).

The posterior median foramen of the fourth ventricle was explored under neuroendoscopy. It was found that the tumor had protruded from the posterior median foramen. The important structures of the latch of medulla oblongata and brain stem were protected, and the tumor was removed in blocks (Fig. 4). Explored the floor of the fourth ventricle under neuroendoscopy. It was found that the lesion was located at the back of the pons. Blocked the outlet of the midbrain aqueduct, separated and removed the lesion. The exploration showed that the cerebrospinal fluid circulation of the fourth ventricle and midbrain aqueduct was unobstructed (Fig. 5).

### Clinical presentation

#### Case 1

A 62-year-old woman presented with dizziness and vomiting for 12 days and was transferred to our hospital. Neurological examination showed difficulty walking, and other exams were normal for cranial nerves, motor/sensory function, coordination, and reflexes. CT imaging showed small hematoma in the right fourth ventricle, middle cerebellar peduncle, and pontine tegmentum. Magnetic resonance imaging (MRI) performed with T1-weighted, T2-weighted and SWAN sequences suggested a cavernous malformation. The preoperative diagnosis was a cavernous malformation in the right middle cerebellar peduncle and pontine tegmentum. After discussion with her family, suboccipital craniotomy was planned.

#### Case 2

A 54-year-old woman presented with numbness in right limb for one year. One year ago, she felt numbness in the right limb and CT scan shown small hemorrhage in the brainstem. After conservative treatment, she was improved and discharged from local hospital. But two weeks ago, she felt numbness in right side and came to hospital again, and CT scan shown rebleeding in the brainstem. Then she was admission to our department and MRI suggested a cavernous malformation in the left pontine tegmentum. After consent of the patient and her family, suboccipital craniotomy with telovelar approach by the neuroendoscopy was prepared.

#### Case 3

A 67-year-old woman with dizziness for 2 months. Brain MRI examination in the local hospital found a lesion on the right side of brainstem, so she was referred to our hospital. Brain MRI + SWI in our hospital showed cavernous hemangioma of the right pontine arm. Communicate the condition and operation plan with the patient and her family, and operate with the consent. The patient recovered well after operation, the symptoms of dizziness disappeared, and there were no obvious complications after discharge.

#### Case 4

A 3-year-old boy with unstable walking for 3 days. Before admission, the child was unstable in walking, fell for many times, and his body tilted to the right. Brain CT of the local hospital showed a lesion on the brainstem. After being transferred to our hospital, the child had rapid heart rate, disturbance of consciousness, vomiting and aspiration, airway obstruction, endotracheal intubation, and other rescue treatment. The emergency brain MRI + enhanced examination showed that the medulloblastoma on the brainstem were possible. The patient was in critical condition. After communicating with the family members and obtaining the consent of the family members, the emergency craniotomy was performed. After the operation, the child was complicated with severe pneumonia and transferred to pediatric ICU for further treatment. Pathological examination showed medulloblastoma. The child’s vital signs were unstable, and the prognosis was very poor. So the family chose to give up treatment and leave the hospital automatically.

#### Case 5

A 32-year-old woman with dizziness and nausea for 3 months. The patient went to the local hospital and underwent brain MRI, which showed that the obstructive hydrocephalus. After referral to our hospital, brain MRI + enhanced examination showed that the lower end of midbrain aqueduct occupied space and the supratentorial hydrocephalus was formed. Communicate the condition and operation plan with patient and her families and operate with the consent. Pathological examination showed pilocytic astrocytoma. The patient recovered well after operation, the symptoms of dizziness and nausea disappeared, there were no obvious complications after discharge, and the hydrocephalus improved after follow-up.

## Results

Among the 5 patients, there were 3 cases of brainstem cavernous hemangioma and 2 cases of brainstem tumor (1 case of medulloblastoma, 1 case of pilocytic astrocytoma). All patients underwent neuroendoscopic telovelar approach to remove the lesions, and the operation was successfully completed. The 4 patients recovered well; the other 1 patient had serious complications of other systems after the operation and discharged automatically.

The symptoms of dizziness and vomiting disappeared immediately after the surgery in the Case 1, 3 and 5, and the numbness was decreased in the Case 2. These four patients’ hospital course and recovery were uneventful. They recovered well and showed no new signs of brainstem or cerebellar dysfunction and were discharged after 2 weeks postoperatively. The Case 4 was discharged automatically with serious complications of other systems after the operation.

## Discussion

Surgical access to lesions in the fourth ventricle may be achieved by utilizing transvermian or telovelar approach. Traditional transvermian approaches require splitting of the inferior vermis to gain better direct access from the posterior direction to the fourth ventricle. This approach inflicts the midline cerebellar structures and has been implicated in postoperative “cerebellar mutism syndromes” [3].

Matsushima et al. [7] firstly described the microsurgical anatomy of the cerebellomedullary fissure and found that it was actually a virtual space existing between the cerebellum and the medulla oblongata that was a natural corridor to the fourth ventricle. By elevating the cerebellar tonsils and opening the cerebellomedullary fissure, the tela choroidea and the inferior medullary velum could offer wide access to the fourth ventricle cavity without the need for splitting the vermis [8]. This technique was applied by other surgeons and has been developed and described as the “telovelar approach” [1, 8, 9]. The telovelar approach can widely expose the fourth ventricle from bottom to top by opening the choroid and inferior medullary sail, without cutting the cerebellar vermis, to reduce the occurrence of postoperative silent syndrome. When using the transparent sheath, we should pay attention to protect the structures of the latch of medulla oblongata and the floor of the fourth ventricle.

Since then, numerous reports of resection of various fourth ventricle tumors, arteriovenous malformations and aneurysms via this approach have been extensively described [1, 3, 9, 10], and it appears that this approach has the potential to become the standard treatment for most lesions of the fourth ventricle with satisfactory results [1]. However, in terms of the vertical working angle of the microscope, it was easy to look from the roof to the floor of the fourth ventricle but was very difficult to assess from caudal to rostral via the telovelar approach. Therefore, this approach was limited in achieving access to the rostral third of the fourth ventricle and middle cerebellar peduncle. Under microscope, a possible maneuver to achieve more favorable working angle to the upper ventricle is to cut the posterior arch of the atlas. Deshmukh et al. found that additional removal of the C1 arch offered a larger working area that contributed to reaching the rostral half of the fourth ventricle [11]. However, it is difficult to access upper fourth ventricle from caudal to rostral without removal posterior arch of the atlas due to the vertical working angle of microscope. Neuroendoscopy has a good degree of freedom in surgery and can reach this area easily. Neuroendoscopy can overcome the limitations of microscope by taking advantage of the freedom achieved during surgery, as the neuroendoscopy can be operated easily from caudal to rostral in the fourth ventricle. Several authors have reported using angled endoscopy assistance for facilitating additional inspection around the anatomic corners and of tumor resection in the fourth ventricle [12], but none full neuroendoscopic telovelar approach was attempted. Our successful cases demonstrate that access to the upper fourth ventricle via the full neuroendoscopic telovelar approach without removing the posterior arch of the atlas is feasibility.

## Conclusions

The telovelar approach has gained popularity as a safe and effective strategy for lesions in fourth ventricular and pons. However, without removing the posterior arch of the atlas, it is difficult to enter the upper part of the fourth ventricle under a microscope. Transcranial neuroendoscopy can effectively compensate for the shortcomings of microscopy, whether used as an auxiliary measure for microsurgery or alone with proficient endoscopic techniques, it will provide greater application in minimally invasive surgery for fourth ventricle and brainstem lesions. By utilizing the excellent degree of freedom of transcranial neuroendoscopy, there is no need to open the posterior arch of the atlas, making the surgery more minimally invasive. However, the sample size of this study is small, and it was completed under the very mature neuroendoscopic technology of our team. Its general safety and practicality still require extensive clinical research validation.

## Data Availability

All data meet the requirements of the journal and can be used by the magazine at will.
